# Intestinal FXR Activation via Transgenic Chimera or Chemical Agonism Prevents Colitis-Associated and Genetically-Induced Colon Cancer

**DOI:** 10.3390/cancers14133081

**Published:** 2022-06-23

**Authors:** Marica Cariello, Roberta Zerlotin, Emanuela Pasculli, Elena Piccinin, Claudia Peres, Emanuele Porru, Aldo Roda, Raffaella Maria Gadaleta, Antonio Moschetta

**Affiliations:** 1 Department of Interdisciplinary Medicine, University of Bari “Aldo Moro”, Piazza Giulio Cesare 11, 70124 Bari, Italy; marica.cariello@uniba.it (M.C.); roberta_zerlotin@libero.it (R.Z.); emanuela.pasculli@uniba.it (E.P.); claudiaperes18@gmail.com (C.P.); 2 National Institute of Biostructures and Biosystems (INBB), 00136 Rome, Italy; emanuele.porru2@unibo.it (E.P.); aldo.roda@unibo.it (A.R.); 3 Department of Basic Medical Science, Neurosciences and Sense Organs, University of Bari “Aldo Moro”, Piazza Giulio Cesare 11, 70124 Bari, Italy; elena.piccinin@uniba.it; 4 Department of Chemistry “Giacomo Ciamician”, University of Bologna, 40126 Bologna, Italy

**Keywords:** Farnesoid X Receptor (FXR), colitis, colon cancer

## Abstract

**Simple Summary:**

Disruption of Bile Acids (BA) regulation with increased BA concentration and modulation or their detergent pro-inflammatory activity has been linked to colorectal cancer (CRC). Farnesoid X Receptor (FXR) is the master regulator of BA homeostasis; FXR is a nuclear receptor that transcriptionally modulates their synthesis, transport and metabolism. In this study, we demonstrated that intestinal FXR activation prevented both inflammation- and genetically-driven colorectal tumorigenesis by modulating BA pool size and composition. This could open new avenues for the therapeutic management of intestinal inflammation and tumorigenesis.

**Abstract:**

The Farnesoid X Receptor (FXR) is the master regulator of Bile Acids (BA) homeostasis orchestrating their synthesis, transport and metabolism. Disruption of BA regulation has been linked to gut-liver axis diseases such as colorectal cancer (CRC). In this study, firstly we examined the role of constitutive activation of intestinal FXR in CRC; then we pre-clinically investigated the therapeutic potential of a diet enriched with a synthetic FXR agonist in two models of CRC (chemically-induced and genetic models). We demonstrated that mice with intestinal constitutive FXR activation are protected from AOM/DSS-induced CRC with a significant reduction of tumor number compared to controls. Furthermore, we evaluated the role of chemical FXR agonism in a DSS model of colitis in wild type (WT) and FXR^null^ mice. WT mice administered with the FXR activating diet showed less morphological alterations and decreased inflammatory infiltrates compared to controls. The FXR activating diet also protected WT mice from AOM/DSS-induced CRC by reducing tumors’ number and size. Finally, we proved that the FXR activating diet prevented spontaneous CRC in APC^Min/+^ mice via an FXR-dependent modulation of BA homeostasis. Our results demonstrate that intestinal FXR activation prevented both inflammation- and genetically-driven colorectal tumorigenesis by modulating BA pool size and composition. This could open new avenues for the therapeutic management of intestinal inflammation and tumorigenesis.

## 1. Introduction

Gut homeostasis is a delicate and very sophisticated balance among the intestinal epithelium, the resident immune system and the gut microbiota. Signals at the crossroad of these three major players ensure the elimination of pathogens and maintenance of self-tolerance. The gastrointestinal tract is constantly exposed to a plethora of potentially harmful triggers-including antigens, toxic molecules, and microorganisms-during our entire life. Disruption of gut homeostasis could lead to uncontrolled chronic inflammation.

Inflammatory bowel disease (IBD) is a chronic intestinal inflammatory disorder characterized by persistent intestinal inflammation, with a global rise in incidence and prevalence especially in Western and newly industrialized countries [1]. In line with this, the incidence of colorectal cancer (CRC) is on the rise paralleling the same geographic pattern [2]. Intriguingly, one of the most important overlapping risk factors for the onset and development of IBD and CRC is the extensive consume of the western high fat diet (HFD). In fact, only 20% of all CRC cases is hereditary [3] while the vast majority is sporadic and driven by environmental factors. Somatic mutation of the adenomatous polyposis coli (APC) gene commonly triggers colorectal carcinogenesis [4], while a germline mutation of APC affects patients with autosomal dominantly inherited familial adenomatous polyposis (FAP) gene [5,6]. Kicked off by diverse triggers such as harmful dietary patterns and the occurrence of chronic inflammation, CRC requires many years to develop through a multistep process, gradually generating derangements of gut physiological processes.IBD is associated with the increased risk of colorectal carcinogenesis proportionally higher to prolonged disease duration, frequent relapse and a concomitant cholangiopathy, namely primary sclerosing cholangitis [7,8,9]. Patients with one of the main IBD phenotype, ulcerative colitis (UC), and associated colonic dysplasia or carcinoma display a higher concentration of fecal bile acids (BA) [10]. Moreover, murine and human studies have linked elevated fecal secondary BA concentration to increased colonic carcinogenesis [11,12], thus identifying secondary BAs as major dietary-related factors in colon carcinogenesis [13,14,15].

BAs are amphipathic molecules synthesized in the liver, stored into the gallbladder and released into the duodenum after food intake where they start their journey along the whole intestinal tract facilitating the absorption of dietary lipids and liposoluble nutrients. BA synthesis is energetically costly; therefore, once they reach the terminal ileum they are actively taken up and re-circulated via the so-called enterohepatic circulation. BAs act as signaling molecules activating the nuclear Farnesoid X receptor (FXR) [16,17,18], a transcription factor highly expressed in the gut-liver axis. FXR operates as the master regulator of BA homeostasis priming the transcription of tissue-specific gene networks orchestrating their synthesis, transport and metabolism (reviewed in [19]). *De novo* hepatic BA synthesis occurs mainly via the classical biosynthetic pathway controlled by negative gut-liver feedback controlling the rate-limiting enzyme converting cholesterol into BA, namely the Cholesterol 7 alpha-hydroxylase (CYP7A1). In the terminal ileum, BA-activated FXR primes the expression of the enterokine FGF15/19 (in mouse and human, respectively) that via the enterohepatic circulation reaches the liver and initiates a phosphorylation cascade which ultimately leads to CYP7A1 repression [20].

An altered BA signaling in the gut-liver axis, and particularly abnormally elevated toxic secondary BAs levels, has been associated with a wide range of severe diseases including chronic intestinal inflammation [21,22,23] and CRC [reviewed in [24,25]. A dysregulation in the BA-dependent modulation of the intestinal stem cell niche via a non-physiological activation of BA-FXR signaling, as it occurs in HFD, may represent a risk factor for CRC onset and progression [26]. A High Fat Diet (HFD) stimulates BA secretion into the intestine and favors the enrichment of specific strains of gut bacteria with enzymatic activity modulating BA metabolism and, in particular, those increasing levels of potentially toxic secondary BAs [27,28]. A HFD in murine models causes a dysregulation of colonic mesenchymal stromal cells, resulting in overexpression of Wnt2b, proliferation and activation of cancer-associated fibroblast-like properties [26]. In addition, population-based studies have shown that subjects who mainly consume a Western diet display elevated concentration of fecal BAs, as do patients diagnosed with colonic carcinomas [29,30].

Several studies have highlighted the role of FXR in maintaining the integrity of the intestinal epithelial barrier, decreasing the pro-inflammatory response [23,31] and controlling bacterial hyperproliferation [32]. Conversely, mice with ablation of FXR display a compromised intestinal epithelial barrier already in the basal state [23,32]. Pharmacological targeting of FXR by the semi-synthetic BA Obeticholic acid, a drug that is already in clinical for the treatment of primary biliary cholangitis (PBC), has been shown to counteract the intestinal inflammatory response in chemically-induced murine models of colitis, beneficial effects that are absent in FXR^−/−^ mice [23]. Furthermore, FXR activation not only ameliorates intestinal inflammation but also IBD-associated CRC. In fact, FXR activation by Nelumal A in mice subjected to a chemical model of colitis and colorectal carcinogenesis attenuates inflammation and oxidative stress and reduces cell proliferation in the colonic mucosa [33]. Several studies have highlighted the crucial role of FXR in intestinal carcinogenesis and the inverse relationship between Fxr expression levels and CRC development [34,35,36,37]. In line with this, Fxr ablation increases susceptibility to chemically-induced colorectal carcinogenesis associated to inflammation [35], while constitutive activation of FXR in colon cancer cells injected in a xenograft model is able to suppress colonic epithelium proliferation and induce a pro-apoptotic gene network, [36]. In addition, FXR expression is greatly reduced in FAP patients and in tumors of Apc^Min/+^ mice spontaneously developing intestinal neoplasia [38,39], and Fxr deficiency in APC^Min/+^ mice results in increased adenoma size and number and is associated with a higher prevalence of tumors in azoxymethane-induced tumorigenesis [35,36]. Moreover, selective activation of intestinal FXR can limit abnormal Lgr5^+^ intestinal stem cell proliferation and curb CRC progression [40]. Thus, strategies aimed at reactivating FXR expression in colon cancer might be helpful in treatment of CRC.

INT-767 is a dual FXR and membrane bile acid receptor TGR5 agonist, a semisynthetic 23-sulfate derivative of the obeticholic acid. We have previously shown that specific FXR activation induced by long-term administration of a diet enriched in INT-767 prevents spontaneous hepatocarcinogenesis in two different murine models of liver cancer, including an inflammation-driven hepatocellular carcinoma [41] via reducing the circulating BA pool size and its hydrophobicity. In this study, we aim to examine the role of FXR activation in both inflammatory- and genetically-driven CRC by administering a diet enriched in INT-767 to mice subjected to experimental colitis and in two models of colorectal carcinogenesis (chemically-induced and genetic).

## 2. Material and Methods

### 2.1. Mice

iVP16 and iVP16-FXR transgenic mice were previously generated in our laboratory [42]. Wild-type (WT) C57BL/6J mice were obtained from Charles River Laboratories [Calco (Lecco), Italy]. Pure strain C57BL/6J Fxr^null^ mice were originally kindly provided by Dr Frank Gonzalez (NIH, Bethesda, MD, USA). Apc^Min/+^ mice were obtained from Jackson laboratory (Bar Harbor, ME, USA). All mice were housed under pathogen-free conditions in a temperature-controlled room (23 °C) on a 12-h light/dark cycle and fed either a standard rodent chow diet or specific rodent diet containing 62.5 mg/Kg of INT-767 (INT-767 diet) and autoclaved tap water *ad libitum*. The Ethical Committee of the University of Bari approved this experimental set-up, which also was certified by the Italian Ministry of Health in accordance with internationally accepted guidelines for animal care.

### 2.2. Colitis Carcinogenesis Model

Two different chemically-induced colitis carcinogenesis model [43] were set-up. In the first experiment, 8 weeks old male iVP16 and iVP16FXR mice were injected intraperitoneally with 12 mg/Kg body weight of azoxymethane (AOM) dissolved in 0.9% NaCl. Five days later, 2.5% dextran sulfate sodium (DSS) was administrated in drinking water over 5 days, followed by 16 days of regular water. This cycle was repeated for a total of 3 times. Subsequently, 8 weeks-old male WT (*n* = 7 mice/group) or Fxr^null^ mice (*n* = 5 mice/group) were randomly divided into 2 experimental arms and fed with either a diet enriched with INT-767 (62.5 mg/Kg) or control diet for 12 weeks. Subsequently, mice were injected intraperitoneally with 12 mg/Kg body weight of azoxymethane (AOM) dissolved in 0.9% NaCl. Five days later, 3.5% dextran sulfate sodium (DSS) was administrated in drinking water over 5 days, followed by 16 days of regular water. This cycle was repeated for a total of 3 times. Mouse individual body weight was recorded every 3 days. After sacrifice, ilea, colon and livers were collected and either snap-frozen or fixed in 10% formalin and subsequently embedded in paraffin. Total number of intestinal tumors was recorded, and we used the mouse number of each group to normalize tumor number. The diameter of each tumor was measured with a caliper.

### 2.3. Colitis Model

For the chemically-induced model of colitis, 8 weeks-old WT or Fxr^null^ mice (*n* = 10 mice per groups) were randomly divided into 2 experimental groups and fed with either a diet enriched with INT-767 (62.5 mg/Kg) or control diet for 5 weeks. Colitis was then induced by administration of 4% (*w*/*v*) dextran sodium sulfate (DSS; molecular mass 36–50 kDa; MP Biochemicals Inc, Amsterdam, The Netherlands) in drinking water for 7 days. Symptoms of colitis were assessed daily. In particular, daily changes in body weight and visible rectal bleeding were recorded. Visible rectal bleeding was scored on a scale from 0 to 5, indicating no (0) to very severe (5) rectal bleeding. Mice were sacrificed on day 7. Ilea, colons and livers were snap-frozen or fixed in 10% formalin (24 h) and embedded in paraffin for downstream analysis.

### 2.4. Intestinal Permeability Assay

In vivo intestinal permeability was assessed in mice on the day of sacrifice. Mice were gavaged with 0.6 mg/g body weight of fluorescein isothiocyanate (FITC)-conjugated dextran (Sigma, St. Louis, MO, USA; molecular mass 3–5 kDa) for 4 h. Blood was collected, and FITC concentrations were measured in plasma with the Microplate fluorometer VICTORTM EnLiteTM (PerkinElmer, Inc., Milan, Italy). Serum fluorescence intensity positively correlates with intestinal permeability.

### 2.5. Adenomatous Polyposis Coli Apc^Min/+^ Mice

4 weeks-old APC^Min/+^ mice were randomly divided into 2 experimental groups and fed with either a specific rodent diet containing 62.5 mg/Kg of INT-767 (Intercept Pharmaceuticals Inc, NY, USA) or control diet for 24 weeks (*n* = 10 APC^Min/+^ CTRL and *n* = 9 APC^Min/+^ INT-767). During the experimental period, individual body weight was recorded every 7 days. Mice were sacrificed at 6 months. Ilea, colons, and livers were snap-frozen or fixed in 10% formalin (24 h) and embedded in paraffin. Total number of intestinal tumors was recorded, and the diameter of each tumor was measured with a caliper.

### 2.6. BA Measurements

Serum BAs were identified and quantified by high-pressure liquid chromatography-electrospray-mass spectrometry/mass spectrometry (HPLC-ES-MS/MS) by optimized methods [41] suitable for use in pure standard solution and serum samples after appropriate clean-up preanalytical procedures. Liquid chromatography analysis was performed using an Alliance HPLC system model 2695 from Waters combined with a triple quadruple mass spectrometer QUATTRO-LC (Micromass; Waters) using an electrospray interface. The analytical column was a Waters XSelect CSH Phenyl-hexyl column, 5 µm, 150 × 2.1 mm, protected by a self-guard column Waters XSelect CSH Phenyl-hexyl 5 µm, 10 × 2.1 mm. BAs were separated by elution gradient mode with a mobile phase composed of a mixture ammonium acetate buffer 15 mM, pH 8.0 (Solvent A) and acetonitrile:methanol = 75:25 *v*/*v* (Solvent B). Chromatograms were acquired using the mass spectrometer in multiple reactions monitoring mode.

### 2.7. RNA Extraction and Quantitative Real Time qRT-PCR Analysis

RNA was isolated from liver and ileum of mice using RNeasy Micro kit (Qiagen, Milano, Italy). cDNA was generated from 4 μg total RNA using High Capacity DNA Archive Kit (Applied Biosystem, Foster City, CA, USA) and following the manufacturer’s instructions. Primers to detect the *mRNA* expression level of each gene were designed using Primer Express software (Applied Biosystem) based on Gene Bank sequence data. *mRNA* expression levels were quantified by qRT-PCR using Power Sybr Green chemistry and normalized to *cyclophilin mRNA* levels for the DSS experiment and *Gapdh* for the AOM-DSS experiments and Apc^Min/+^ mice. Validated primers for qRT-PCR are available upon request. Real time qPCR dataare calculatedusing the Ct of basal condition or control as calibrator. Then, we represent relative quantification as mean of replicates.

### 2.8. Histology and Immunohistochemistry

Colon specimens were snap-frozen or fixed in 10% formalin (24 h), dehydrated and embedded in paraffin. Tumor were stored in ethanol 50% and for this reasonunfortunately we are not able to provide entire tumor pictures.Distal colon sections (5 µm) were stained with hematoxylin and eosin (H&E) according to manufacturer’s instructions. Stained slides were scanned on an AperioScanScope AT. For the DSS experiment, the histopathological scoring of inflammation was performed using an established semiquantitative score ranging from 0 to 6 based on infiltration of inflammatory cells and epithelial damage (1 = few inflammatory cells, no epithelial degeneration; 2 = mild inflammation, few signs of epithelial degeneration; 3 = moderate inflammation, few epithelial ulcerations; 4 = moderate to severe inflammation, ulcerations in 25–50% of the tissue section; 5=moderate to severe inflammation, large ulcerations in >50% of the tissue section; 6=severe inflammation and ulcerations of >75% of the tissue section) [44]. Distal colonic sections were also stained for PCNA. Briefly, sections were subjected to antigen retrieval by boiling the slides in sodium citrate pH 6 (Sigma Aldrich, Milan, Italy) for 15 min. Sections were permeabilized in phosphate-buffered saline (PBS) with 0.25% TritonX-100 for 5min and were sequentially incubated for 10 min at room temperature in protein blocking solution (Dako, Glostrup, Denmark) and overnight at 4 °C with the primary antibodies (anti-pcna, Santa Cruz Biotechnology, Santa Cruz, CA, USA). Sections were washed 15 min in PBS and incubated for 25min at room temperature with DAKO real EnVision detection system Peroxidase/DAB+ (Dako, Glostrup, Denmark) according to manufacturer’s instruction. After washing in PBS, the peroxidase reaction was initiated by incubation with DAB (Dako, Glostrup, Denmark). Cover slips were mounted with Permount and evaluated under a light microscope. Image processing was performed using Image J software. For each sample, 10 representative images were taken with a 20× objective. The percentage of stained area/total area was measured. Values from all consecutive images for each sample were averaged. For negative controls, 1% non-immune serum in PBS replaced the primary antibodies.

### 2.9. Statistical Analysis

All results are expressed as mean ± standard error of the mean (SEM). Statistical analysis was performed using GraphPad Prism software (v5.0; GraphPad Software Inc., San Diego, CA, USA). Comparisons of two groups were performed using a Student’s t two tailed test. A *p* value of <0.05 was considered significant.

## 3. Results

### 3.1. Intestinal Constitutive FXR Activation Protects from AOM/DSS-Induced Colorectal Cancer

**Figure 1:**
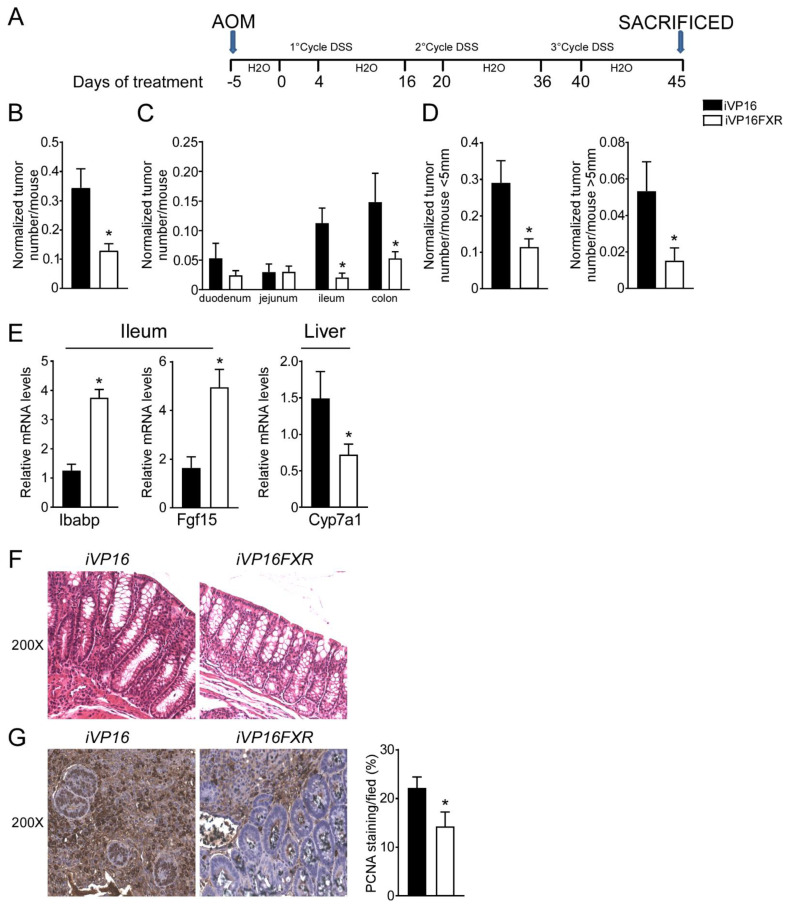
Intestinal constitutive FXR activation protects from AOM/DSS-induced colorectal cancer. (**A**) Schematic representation of AOM/DSS administration. AOM (12 mg/Kg) is injected on day 0. 5 days later, DSS solution is administered to mice in drinking water. 5 days of DSS treatment is followed by 16 days of drinking water. This cycle was repeated a total of 3 times. (**B**) Total number of tumors was counted in iVP16 and iVP16FXR mice. The diameter of each tumor was measured. Normalized tumor number/mouse of iVP16 and iVP16FXR mice (*n* = 13 iVP16 and *n* = 20 iVP16FXR). (**C**) The entire length of the intestine was analyzed for tumor formation (*n* = 13 iVP16 and *n* = 20 iVP16FXR). (**D**) Normalized number of tumors <5 mm and >5 mm per mouse from intestine of iVP16 and iVP16FXR mice (*n* = 13 iVP16 and *n* = 20 iVP16FXR). (**E**) Gene expression analysis of FXR target genes in iVP16 and iVP16FXR mice. Cyclophilin was used as a housekeeping gene to normalize data (*n* = 13 iVP16 and *n* = 20 iVP16FXR). (**F**) Histology of colonic specimens of iVP16 and iVP16FXR mice was assessed by H&E staining and was observed by light microscopy (magnification, 200×) (*n* = 5 iVP16 and *n* = 5 iVP16FXR). Representative specimens are shown. (**G**) Paraffin-embedded tumor specimens from iVP16 and iVP16FXR mice were immunoassayed with anti-PCNA antibody (200×-magnification) (*n* = 5 iVP16 and *n* = 5 iVP16FXR). Representative specimens are shown. PCNA staining per field was quantified by ImageJ software and reported as percentage per field. Results are the mean ± SEM, * *p* ≤ 0.05 Statistical significance was assessed by Student’s *t*-test (* *p* < 0.05).

A role for FXR in intestinal carcinogenesis has emerged in both mouse models and humans [11,12]. To study whether constitutive intestinal Fxr activation protects from intestinal tumorigenesis, iVP16-Fxr mice have been subjected to a model of inflammation-driven colorectal tumorigenesis. AOM-DSS experimental treatment in iVP16 mice caused tumor development (Figure 1A–D), characterized by a progressively increasing number of tumors moving along the different segments of the intestinal tract (Figure 1C). Strikingly, intestinal constitutive expression of FXR prevented intestinal tumorigenesis and over 60% reduction in tumor number per mouse was observed compared to control mice (Figure 1B). Furthermore, stratifying tumors according to their size, a striking decrease in tumor number with a diameter either smaller or bigger than 5 mm was recorded in iVP16FXR compared to controls (Figure 1D). To demonstrate constitutive activation of Fxr, ileal and hepatic target genes expression was analyzed. In this case, iVP16FXR mice displayed an upregulation of ileal IBABP compared to control mice (Figure 1E). In addition, iVP16FXR displayed an increased expression of the intestinal enterokine Fgf15 which, in turn led to hepatic downregulation of Cyp7a1 (Figure 1E). Histological and immunohistochemical analysis revealed protection from the inflammation-driven intestinal carcinogenesis. In particular, H&E staining of colonic section showed that intestinal constitutive activation of Fxr preserved the intestinal epithelial morphology, compared to iVP16 mice (Figure 1F). PCNA immunostaining has been used to mark the proliferative status of colonic cells. AOM-DSS experimental treatment promoted proliferation of colonic cells, which was almost 40% lower in iVP16Fxr mice compared to control mice (Figure 1G).

### 3.2. A Short-Term Administration of a Diet Enriched in INT-767 Activates FXR-Dependent Orchestration of BA Synthesis in the Gut-Liver Axis

**Figure 2:**
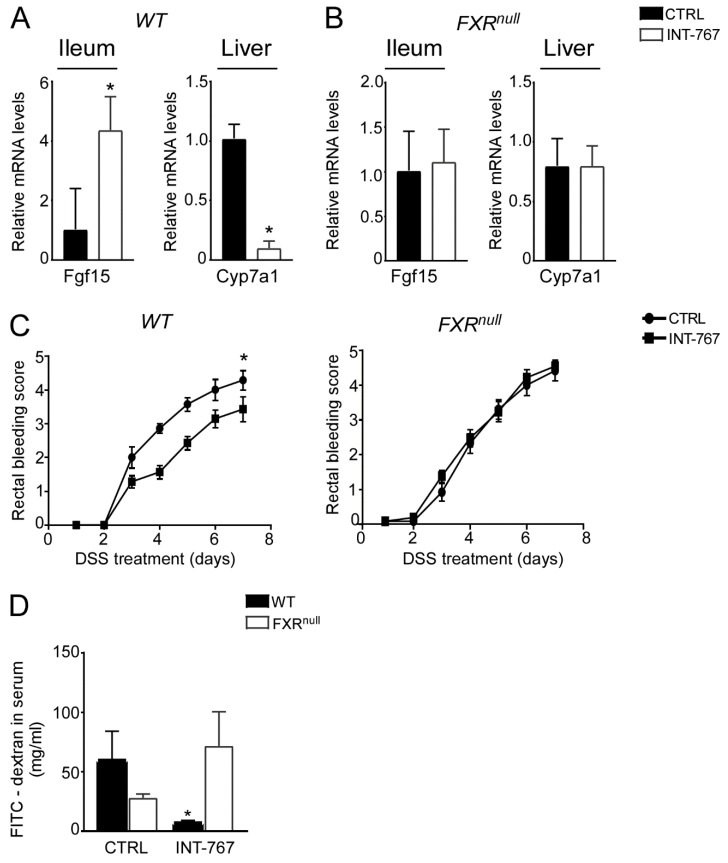
INT-767 protects from DSS-induced colitis via FXR activation. Gene expression analysis of FXR target genes in (**A**) WT and (**B**) Fxr^null^ mice fed with or without INT-767 (*n* = 7 WT CTRL and *n* = 7 WT INT-767; *n* = 6 Fxr^null^ CTRL and *n* = 7 Fxr^null^ INT-767). Cyclophilin was used as a housekeeping gene to normalize data. (**C**) Visible rectal bleeding score in WT and Fxr^null^ mice fed with or without INT-767. (**D**) In vivo intestinal permeability measurement after DSS-treatment in WT and Fxr^null^ mice assessed through FITC administration. All values are represented as means ± SEM (*n* = 7 WT CTRL and *n* = 7 WT INT-767; *n* = 7 Fxr^null^ CTRL and *n* = 7 Fxr^null^ INT-767). Statistical significance was assessed by Student’s *t*-test (* *p* < 0.05).

We have previously shown an INT-767-dependent FXR activation when administered long term (12–14 months) in a rodent diet [41]. To study the therapeutic potential of the FXR-agonist INT-767 in intestinal inflammation in a preventive fashion, a diet-enriched with this compound was administered to mice for 5 weeks prior DSS to evaluate FXR activation via a short-term administration of INT-767 in a rodent diet. We assessed FXR transcriptional activation in the gut-liver axis in our short-term dietetic protocol. In the ileum, WT mice administered with INT-767 displayed a significant up-regulation of FGF15 expression. This, in turn, translated into a down-regulation of Cyp7a1 in the liver. INT-767-dependent FXR activation did not occur in Fxr^null^ mice (Figure 2A,B).

### 3.3. INT-767 Protects from DSS-Induced Colitis via FXR Activation

**Figure 3:**
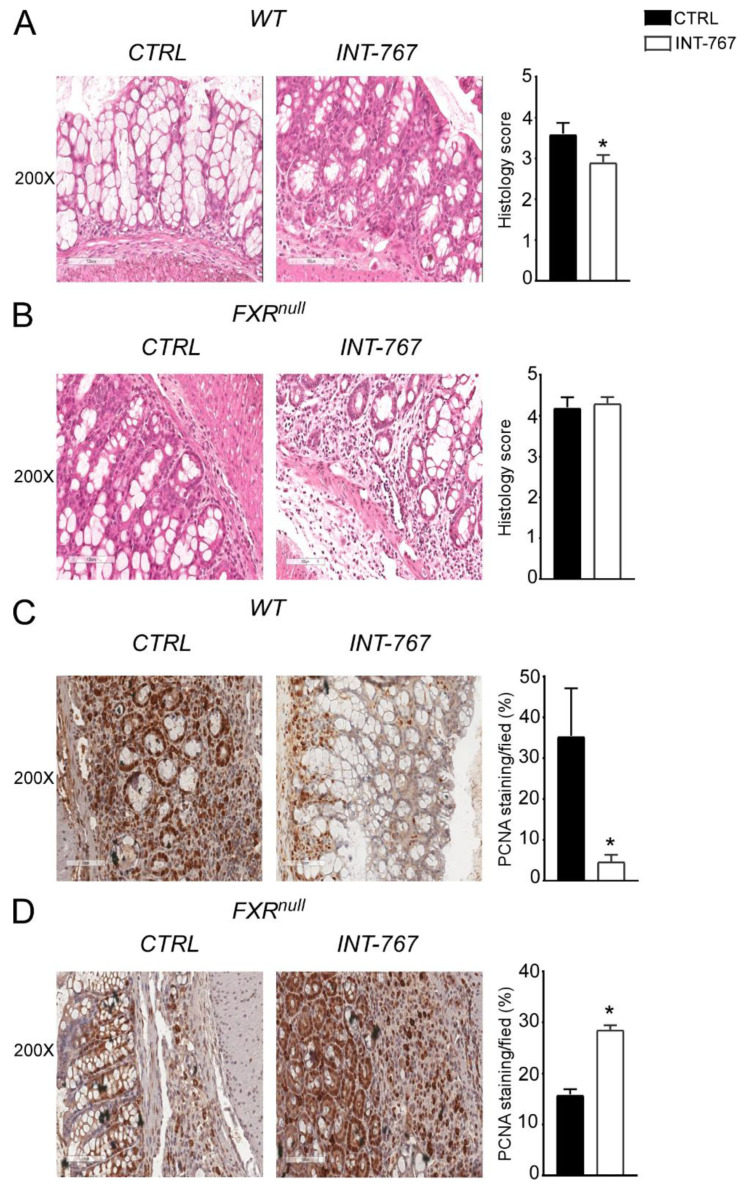
INT-767 protects from DSS-induced colitis. Histology of colonic specimens of (**A**) WT and (**B**) Fxr^null^ mice fed with or without INT-767 was assessed by H&E staining and was observed by light microscopy (magnification, 200×; *n* = 5 WT CTRL and *n* = 5 WT INT-767; *n* = 5 Fxr^null^ CTRL and *n* = 5 Fxr^null^ INT-767). Representative specimens are shown. Histology score of inflammation was calculated using an established semiquantitative score ranging from 0 to 6 based on infiltration of inflammatory cells and epithelial damage. Paraffin-embedded tumor specimens from (**C**) WT and (**D**) Fxr^null^ mice fed with or without INT-767were immunoassayed with anti-PCNA antibody (200× magnification; *n* = 5 WT CTRL and *n* = 5 WT INT-767; *n* = 5 Fxr^null^ CTRL and *n* = 5 Fxr^null^ INT-767). Representative specimens are shown. PCNA staining per field was quantified by ImageJ software and reported as percentage per field. Results are the mean ± SEM. Statistical significance was assessed by Student’s *t*-test (* *p* < 0.05).

After 5 weeks of INT-767-enriched diet feeding, mice were challenged with 4% DSS to induce colitis. INT-767-enriched diet protected WT mice from rectal bleeding, compared to mice administered with the control diet. Conversely, no changes in rectal bleeding were observed in Fxr^null^ mice with or without INT-767-enriched diet (Figure 2C). Interestingly, colon length of Fxr^null^ mice was significantly lower compared to WT mice, regardless of the treatment (data not shown). The INT-767-dependent protection of the colonic morphological structure suggested a preservation of the intestinal epithelial barrier integrity. To assess this in vivo, a FITC-dextran based intestinal epithelial assay was performed. DSS treatment compromised the integrity of the gut barrier in both WT and Fxr^null^ mice. However, WT mice, but not Fxr^null^ mice, fed with INT-767-enriched diet displayed a remarkable reduction in intestinal permeability compared to control mice, as indicated by an almost completely abolishment of plasma level of FITC-conjugated dextran (Figure 2D). Histological analysis on sections stained with H&E indicated that DSS-induced colitis was associated with a severe disruption of the intestinal epithelial layer and acute inflammatory infiltrates in WT mice administered with the control diet. Conversely, WT mice administered with INT-767-enriched diet showed significantly less morphological alterations and decreased inflammatory infiltrates. A histopathological score method was applied and quantification of histological disease index is shown (Figure 3A,B). No significant changes were observed in Fxr^null^ mice administered with either diet.

### 3.4. INT-767 Protects Wild-Type Mice from AOM/DSS-Induced Colorectal Cancer via FXR Activation

**Figure 4:**
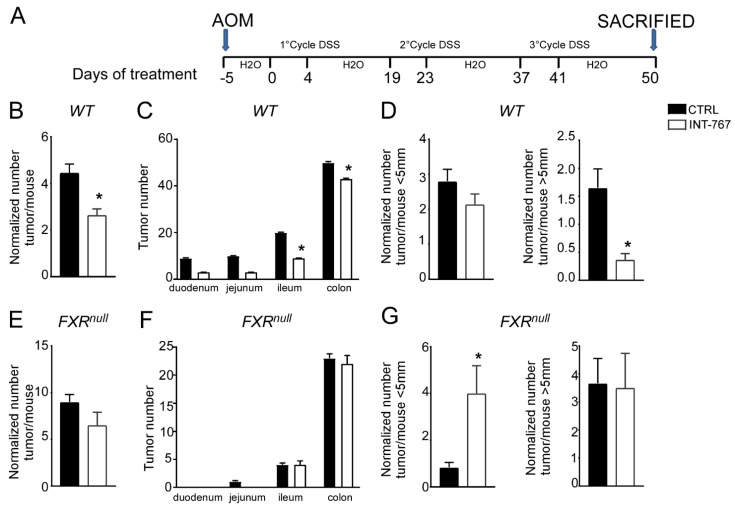
INT-767 protects WT mice from AOM/DSS-induced colorectal cancer. (**A**) Schematic representation of AOM/DSS administration. AOM (12 mg/Kg) is injected on day 0. 5 days later, DSS solution is administered to mice in drinking water. 5 days of DSS treatment is followed by 16 days of drinking water. This cycle was repeated a total of 3 times. (**B**) Total number of tumors was counted in WT mice fed with or without INT-767 diet. The diameter of each tumor was measured (*n* = 7 WT CTRL and *n* = 7 WT INT-767). (**C**) The entire length of the intestine was analyzed for tumor formation (*n* = 7 WT CTRL and *n* = 7 WT INT-767). (**D**) Normalized number of tumors <5 mm and >5 mm per mouse from intestine of WT mice fed with or without IN-767 (*n* = 7 WT CTRL and *n* = 7 WT INT-767). (**E**) Total number of tumors was counted in Fxr^null^ mice fed with or without INT-767 diet. The diameter of each tumor was measured (*n* = 7 Fxr^null^ CTRL and *n* = 7 Fxr^null^ INT-767). (**F**) The entire length of the intestine was analyzed for tumor formation (*n* = 7 Fxr^null^ CTRL and *n* = 7 Fxr^null^ INT-767). (**G**) Normalized number of tumors <5 mm and >5 mm per mouse from intestine of Fxr^null^ mice fed with or without IN-767 (*n* = 7 Fxr^null^ CTRL and *n* = 7 Fxr^null^ INT-767). All results are the mean ± SEM. Statistical significance was assessed by Student’s *t*-test (* *p* < 0.05).

Clinical and experimental evidence have shown that chronic intestinal inflammation plays a crucial role in colorectal carcinogenesis. Indeed, patients affected by IBD present an increased risk of developing intestinal tumors. In these patients, colitis-associated cancer risk correlates with the severity and duration of active disease [9]. Moreover, studies in rodent models of intestinal cancer and human epidemiologic studies have linked elevated fecal concentration of secondary BAs to an increased incidence of colorectal cancer [11,12]. In the DSS model described in Figure 2 and Figure 3, the inflammation-dependent proliferating capacity was assessed by analyzing protein expression of a regulator of cell cycle progression, PCNA, in the distal colon of mice subjected to the chemically-induced colitis. DSS promoted proliferation in both WT and Fxr^null^ mice administered with control diet. Conversely, WT mice administered with INT-767-enriched diet displayed 70% less PCNA staining compared to the control group, while no changes were recorded in Fxr^null^ mice (Figure 3C,D). Therefore, we moved on to a chemically-induce inflammation driven intestinal neoplasia model and studied whether the INT-767-enriched diet has a role in protecting from colorectal tumorigenesis via pharmacological Fxr activation. Thus, we performed an intraperitoneal injection of AOM followed by three subsequent administrations of DSS (Figure 4A). AOM-DSS experimental treatment in WT mice fed with the control diet caused tumor development (Figure 4B), characterized by a progressively increasing number of tumors moving along the different segments of the intestinal tract (Figure 4C). INT-767 administration in WT mice counteracted this and a reduction in tumor number per mouse was observed (Figure 4B,C). Notably, stratifying the tumor/mouse according to tumor size, a striking decrease in tumor number with a diameter larger than 5mm was recorded for WT mice fed with INT-767-enriched diet compared to controls (Figure 4D). No differences were found in Fxr^null^ mice (Figure 4E–G).

**Figure 5:**
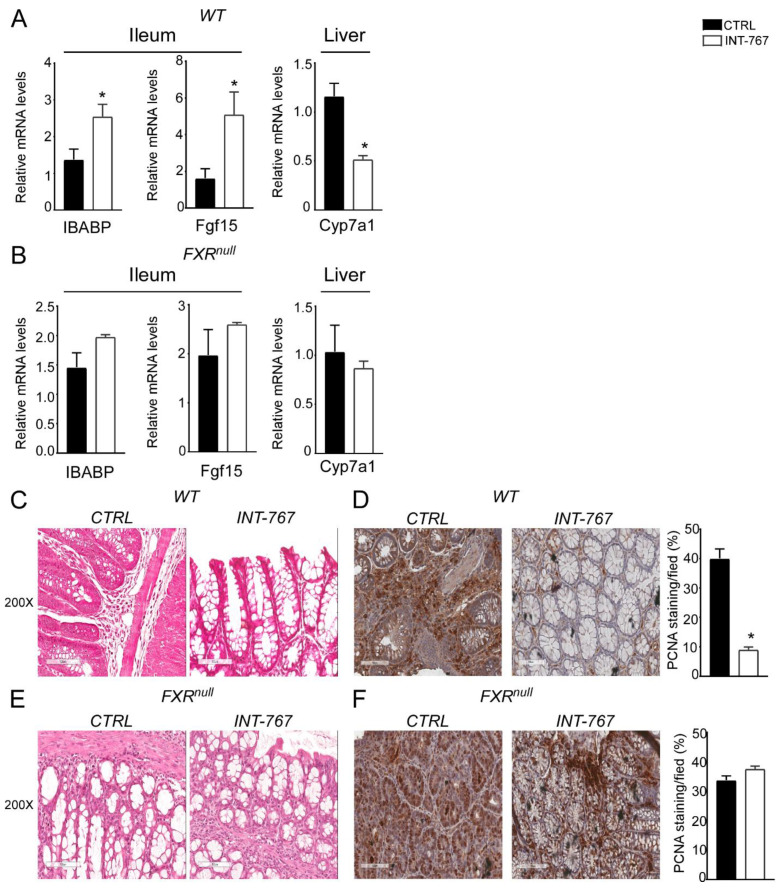
INT-767 protects WT mice from AOM/DSS-induced colorectal cancer via FXR activation.Gene expression analysis of FXR target genes in WT (**A**) and Fxr^null^ (**B**) mice fed with or without INT-767 (*n* = 7 WT CTRL and *n* = 6 WT INT-767; *n* = 4 Fxr^null^ CTRL and *n* = 4 Fxr^null^ INT-767). Cyclophilin was used as a housekeeping gene to normalize data. Histology of colonic specimens of (**C**) WT and (**E**) Fxr^null^ mice fed with INT-767 or control diet was assessed by H&E staining and was observed by light microscopy (magnification, 200×; *n* = 5 WT CTRL and *n* = 5 WT INT-767; *n* = 5 Fxr^null^ CTRL and *n* = 5 Fxr^null^ INT-767). Representative specimens are shown. Paraffin-embedded tumor specimens from (**D**) WT and (**F**) Fxr^null^ mice fed with INT-767 or control diet were immunoassayed with anti-PCNA antibody (200× magnification; *n* = 5 WT CTRL and *n* = 5 WT INT-767; *n* = 5 Fxr^null^ CTRL and *n* = 5 Fxr^null^ INT-767). Representative specimens are shown. PCNA staining per field was quantified by ImageJ software and reported as percentage per field. All results are expressed as mean ± SEM (*p* < 0.05). Statistical significance was assessed by Student’s *t*-test (* *p* < 0.05).

To verify Fxr activation via INT-767 oral administration, we analyzed intestinal and hepatic Fxr target genes. In the ileum of WT mice, INT-767 activated Fxr to induce the expression of IBABP compared to control diet (Figure 5A). Moreover, activated Fxr in the ileum induced the expression of Fgf15 which, in turn, resulted in Cyp7a1 hepatic downregulation (Figure 5A). Conversely, no hepatic nor intestinal Fxr target gene expression was induced by INT-767 in Fxr^null^ mice (Figure 5B).

To explore INT-767-mediated protection from inflammation-driven intestinal carcinogenesis at the histological level, H&E staining of colonic sections was performed. Microscopic analysis showed that INT-767-dependent FXR activation preserves the intestinal epithelial morphology in WT mice compared to vehicle diet fed mice (Figure 5C). No differences were observed in Fxr^null^ mice (Figure 5E). In addition, to evaluate the proliferative status of colonic cells, PCNA immunostaining was performed. The inflammation-driven carcinogenesis induced by the AOM-DSS treatment increases colonocytes proliferative ability. This was counteracted by INT-767-enriched diet in WT mice (Figure 5D). On the contrary, no decrease of PCNA signal was observed in INT-767-fed Fxr^null^ mice compared to control diet (Figure 5F). Taken together, these data demonstrate that short-term administration of a diet enriched with INT-767 promotes anti-inflammatory and anti-proliferative effects in the intestine via Fxr activation.

### 3.5. INT-767 Prevents Spontaneous Colorectal Carcinogenesis in Apc^min/+^ Mice via Fxr-Dependent Orchestration of Ba Homeostasis

**Figure 6:**
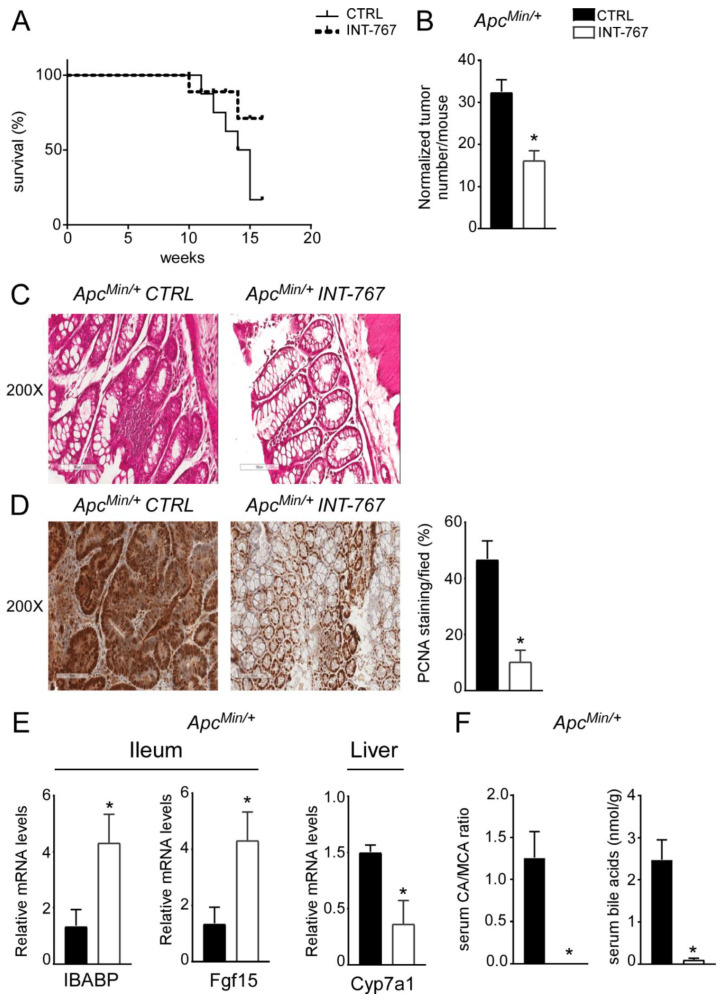
INT-767 prevents spontaneous colorectal carcinogenesis in APC^Min/+^ mice via FXR-dependent orchestration of BA homeostasis. (**A**) Mortality rate in Apcmin/+ mice fed with IN-767 compared to APC^Min/+^ mice fed with chow diet (*n* = 10 APC^Min/+^ CTRL and *n* = 9 APC^Min/+^ INT-767). (**B**) Normalized number of tumors was evaluated in APC^Min/+^ mice fed with or without INT-767 diet (*n* = 4 APC^Min/+^ CTRL and *n* = 7 APC^Min/+^ INT-767). (**C**) Histology was assessed by hematoxylin and eosin (H&E) staining and was observed by light microscopy (magnification, 200×; *n* = 4 APC^Min/+^ CTRL and *n* = 4 APC^Min/+^ INT-767) in APC^Min/+^ mice fed with or without INT-767 diet. Representative specimens are shown. (**D**) Paraffin-embedded tumor specimens from APC^Min/+^ mice fed with or without INT-767 diet were immunoassayed with anti-PCNA antibody (200× magnification; *n* = 4 APC^Min/+^ CTRL and *n* = 4 APC^Min/+^ INT-767). Representative specimens are shown. PCNA staining per field was quantified by ImageJ software and reported as percentage per field. (**E**) Gene expression analysis of FXR target genes in APC^Min/+^ mice fed with or without INT-767 diet. Cyclophilin was used as a housekeeping gene to normalize data (*n* = 4 APC^Min/+^ CTRL and *n* = 4 APC^Min/+^ INT-767). (**F**) Serum BA levels in APC^Min/+^ mice fed with or without INT-767 diet (*n* = 4 APC^Min/+^ CTRL and *n* = 4 APC^Min/+^ INT-767). All values are represented as means ± SEM. Statistical significance was assessed by Student’s *t*-test (* *p* < 0.05).

About 20% of colorectal cancer cases have a genetic base [3] as it occurs in FAP patients, a syndrome caused by a mutation in the APC gene. Typically, FAP patients display an early development of multiple polypoid tumors already at a young age. A murine genetic model of this disease, the Apc^Min/+^ mouse, is available [38,39]. Apc^Min/+^ mice carry a truncated mutation of the tumor suppressor APC gene which causes spontaneous development of intestinal tumors in mice, starting at 4 months of age. In Apc^Min/+^ mice, tumors spontaneously develop from precursor lesions, known as aberrant crypt foci, to polypoid adenomas and they appear primarily in the small intestine and at later stages within the colon. We have previously shown that Fxr expression is lower in both Apc^Min/+^ mice and in FAP patients’ tumors compared to the macroscopically normal adjacent mucosa [36]. Moreover, lack of FXR in both Apc^Min/+^ and in the AOM-DSS model of inflammation-driven intestinal tumorigenesis, leads to early mortality and faster tumor progression. On this background, the potential effect of FXR activation via administration of a diet enriched in INT-767 has also been studied in Apc^Min/+^ mice. First of all, mortality assessment showed that Apc^Min/+^ mice fed with INT-767 had 70% reduction in mortality rate compared to mice fed with the control diet (Figure 6A). Furthermore, a significant reduction in normalized tumor number in Apc^Min/+^ mice fed with INT-767 diet compared to control group was observed (Figure 6B). Subsequently, histological (H&E) and immunohistochemical (PCNA) analyses were performed on colonic sections. H&E staining showed a preserved intestinal morphology compared to mice fed with control diet (Figure 6C). Moreover, a lower PCNA signals was observed in Apc^Min/+^ mice fed with INT-767 diet compared to the control group (Figure 6D). To assess whether the antitumoral effect of INT-767 was induced via Fxr activation in the gut-liver axis and the consequent reshape of BA homeostasis, FXR target gene expression and serum BA pool size and composition were analyzed. In the ileum, a significant up-regulation of Ibabp was observed in Apc^Min/+^ fed with INT-767-enriched diet compared to controls (Figure 6E). Fgf15 expression was also induced by INT-767-dependent activation of Fxr, which in turn resulted in hepatic downregulation of Cyp7a1 (Figure 6E). Consequently, this caused a marked decrease in serum BA levels in mice fed with INT-767 diet and a shift of serum BA composition to a more hydrophilic profile due to the enrichment of β-muricholic acid (MCA) in the BA pool, as indicated in the graph reporting CA/MCA ratio, compared to controls (Figure 6F). We do not provide the difference in Cyp7a1 protein levels, and we recognized this is a limitation of the study although the reduction of serum BA concentration and serum CA/MCA ratio represents its functional readout.

## 4. Discussion

Colorectal cancer was rather rare in 1950 but has progressively reached a predominant incidence in Western countries, now accounting for approximately 10% of cancer-related mortality [45]. Dietary factors, and especially high-fat Western-like diets, are long recognized as risk factors involved in the etiology of colorectal cancer [13,14,15,25,45,46]. Experiments in rodent models and observational human data have shown that the tumor-promoting activity of a Western-style diet is associated with increased colonic BA concentrations and higher fecal BA levels. Chronically elevated levels of BAs have been associated to the disruption of gut homeostasis and uncontrolled chronic intestinal inflammation. The association between IBD and colorectal carcinogenesis has been thoroughly established and both pathologies are on the rise in Western countries, and abnormally elevated levels of fecal BA have been linked to ulcerative colitis and colitis-associated colonic dysplasia or carcinoma [10,11,12]. Accordingly, population-based studies have shown that subjects who mainly consume a Western-type diet display elevated levels of fecal BAs, as do patients diagnosed with colonic carcinomas [29,30]. Despite their extremely important role in intestinal physiology, BA levels must be tightly regulated in the gut-liver axis due to their cytotoxic detergent properties.

The nuclear receptor FXR is the master regulator of BA homeostasis. Pharmacological FXR activation and/or therapeutic exploitation of the enterokine FGF19 is known to attenuate not only intestinal inflammation [22,23,31,32,33], but also hepatocellular carcinoma [41,47], by decreasing circulating BA levels and modulating the BA pool size and composition to a more hydrophilic one. Moreover, FXR expression is reduced in human intestinal cancer samples [36] and FXR deficiency in mice promotes inflammation, cell proliferation and intestinal carcinogenesis [35,36] as well as hepatocellular carcinoma [48,49], due to the lack of the negative feedback loop controlling BA homeostasis. However, when Fxr is constitutively activated in the intestine, as seen in iVP16-Fxr mice, BA homeostasis can be re-established through the Fxr-Fgf15 axis, even in the absence of hepatic FXR. It has recently been shown that FXR activation by Nelumal A in murine experimental model of intestinal inflammation and cancer attenuates inflammation and cell proliferation in the colonic mucosa [33]. Intriguingly, the FXR agonist Obeticholic acid has been shown to inhibit cell proliferation and invasion by repressing proliferative pathways in colon cancer cell lines and in a xenograft tumor model [50], and the synthetic FXR ligand GW4064 acts synergistically with the chemiotherapic agent oxaliplatin to inhibit colon cancer cells growth and their colony formation capabilities [51]. Our study shows, for the first time, that intestinal constitutive FXR activation also prevents inflammation-driven colorectal tumorigenesis via suppression of Cyp7a1-dependent BA synthesis. In line with this observation, colitis and inflammation-associated intestinal cancer could also be hindered via specific pharmacological FXR activation by INT-767-enriched diet, previously shown to protect from the spontaneous development of liver tumor [41] due to abnormal accumulation of hepatic BAs [48,49,52]. Blocking TNFα in mice subjected to DSS and AOM-DSS protocols prevents mucosal ulcer development and reduces colorectal cancer associated with chronic colitis [53]. However, in our models, despite attenuation of the inflammatory signaling, as shown by H&E staining, a decrease in TNFα expression was not observed (data not shown). Intriguingly, INT-767-enriched diet administered to mice carrying a germline mutation of the Apc gene, where inflammation is not the major drive of the spontaneous onset of intestinal cancer, corroborates the finding that, more than the mere direct attenuation of inflammatory pathways, INT-767-dependent Fxr activation, and the associated Fgf15 prompt, inhibits hepatic BA synthesis as demonstrated by Cyp7a1 decrease. Moreover, despite the fact that BA levels in the intestine and tumor tissues have not been measured, the decrease of circulating BAs and modulation of their composition is responsible for the observed Fxr-dependent anti-tumorigenic capacity. In conclusion, attenuation of the inflammatory signals and suppression of proliferative prompts occur via inhibition of de novo BA synthesis.

Great advancements have been made in early screening, adjuvant therapies and surgical techniques in both primary and metastatic CRC [45], however, the mortality rate is still high for CRC as specific therapies are still lacking. In addition, beside the genetic signature of each CRC patients, the multitude of environmental factors boosting its incidence is mirrored by the heterogeneity of its multiple molecular pathogenesis. Further research is imperative to unfold the association between the bileome and gut microbiota in chronic intestinal inflammation and CRC, and to understand how the nutritional status of a given patient could impact disease incidence and prognosis.

## 5. Conclusions

Our findings further strengthen the indication of FXR-FGF19-based therapies in the clinical management of chronic intestinal inflammation and CRC in patients with concomitant dysregulation of BA homeostasis, possibly due to unhealthy eating patterns leading to intestinal dysbiosis and comorbidities, such as obesity and type 2 diabetes. Moreover, the clinical exploitation of FXR in patients with liver diseases is now a reality and could definitely increase the chance of a smooth translation of our findings to a subset of IBD and CRC patients.

## Data Availability

The data presented in this study are all contained within this publication.

## References

[1] GBD 2017 Disease and Injury Incidence and Prevalence Collaborators (2020). The global, regional, and national burden of inflammatory bowel disease in 195 countries and territories, 1990–2017: A systematic analysis for the Global Burden of Disease Study 2017. Lancet Gastroenterol. Hepatol..

[2] Ferlay J., Colombet M., Soerjomataram I., Parkin D.M., Pineros M., Znaor A., Bray F. (2021). Cancer statistics for the year 2020: An overview. Int. J. Cancer.

[3] Rustgi A.K. (2007). The genetics of hereditary colon cancer. Genes Dev..

[4] Powell S.M., Zilz N., Beazer-Barclay Y., Bryan T.M., Hamilton S.R., Thibodeau S.N., Vogelstein B., Kinzler K.W. (1992). APC mutations occur early during colorectal tumorigenesis. Nature.

[5] Kinzler K.W., Nilbert M.C., Su L.K., Vogelstein B., Bryan T.M., Levy D.B., Smith K.J., Preisinger A.C., Hedge P., McKechnie D. (1991). Identification of FAP locus genes from chromosome 5q21. Science.

[6] Kinzler K.W., Nilbert M.C., Vogelstein B., Bryan T.M., Levy D.B., Smith K.J., Preisinger A.C., Hamilton S.R., Hedge P., Markham A. (1991). Identification of a gene located at chromosome 5q21 that is mutated in colorectal cancers. Science.

[7] Khaderi S.A., Sussman N.L. (2015). Screening for malignancy in primary sclerosing cholangitis (PSC). Curr. Gastroenterol. Rep..

[8] Lazaridis K.N., LaRusso N.F. (2016). Primary Sclerosing Cholangitis. N. Engl. J. Med..

[9] Ullman T.A., Itzkowitz S.H. (2011). Intestinal inflammation and cancer. Gastroenterology.

[10] Hill M.J., Melville D.M., Lennard-Jones J.E., Neale K., Ritchie J.K. (1987). Faecal bile acids, dysplasia, and carcinoma in ulcerative colitis. Lancet.

[11] Bianchini F., Caderni G., Dolara P., Fantetti L., Kriebel D. (1989). Effect of dietary fat, starch and cellulose on fecal bile acids in mice. J. Nutr..

[12] Reddy B.S., Wynder E.L. (1977). Metabolic epidemiology of colon cancer. Fecal bile acids and neutral sterols in colon cancer patients and patients with adenomatous polyps. Cancer.

[13] Caderni G., Bianchini F., Dolara P., Kriebel D. (1989). Proliferative activity in the colon of the mouse and its modulation by dietary starch, fat, and cellulose. Cancer Res..

[14] Reddy B.S. (1981). Diet and excretion of bile acids. Cancer Res..

[15] Reddy B.S. (1981). Dietary fat and its relationship to large bowel cancer. Cancer Res..

[16] Makishima M., Okamoto A.Y., Repa J.J., Tu H., Learned R.M., Luk A., Hull M.V., Lustig K.D., Mangelsdorf D.J., Shan B. (1999). Identification of a nuclear receptor for bile acids. Science.

[17] Parks D.J., Blanchard S.G., Bledsoe R.K., Chandra G., Consler T.G., Kliewer S.A., Stimmel J.B., Willson T.M., Zavacki A.M., Moore D.D. (1999). Bile acids: Natural ligands for an orphan nuclear receptor. Science.

[18] Wang H., Chen J., Hollister K., Sowers L.C., Forman B.M. (1999). Endogenous bile acids are ligands for the nuclear receptor FXR/BAR. Mol. Cell.

[19] Gadaleta R.M., Cariello M., Sabba C., Moschetta A. (2015). Tissue-specific actions of FXR in metabolism and cancer. Biochim. Biophys. Acta.

[20] Inagaki T., Choi M., Moschetta A., Peng L., Cummins C.L., McDonald J.G., Luo G., Jones S.A., Goodwin B., Richardson J.A. (2005). Fibroblast growth factor 15 functions as an enterohepatic signal to regulate bile acid homeostasis. Cell Metab..

[21] Duboc H., Rajca S., Rainteau D., Benarous D., Maubert M.A., Quervain E., Thomas G., Barbu V., Humbert L., Despras G. (2013). Connecting dysbiosis, bile-acid dysmetabolism and gut inflammation in inflammatory bowel diseases. Gut.

[22] Gadaleta R.M., Garcia-Irigoyen O., Cariello M., Scialpi N., Peres C., Vetrano S., Fiorino G., Danese S., Ko B., Luo J. (2020). Fibroblast Growth Factor 19 modulates intestinal microbiota and inflammation in presence of Farnesoid X Receptor. EBiomedicine.

[23] Gadaleta R.M., van Erpecum K.J., Oldenburg B., Willemsen E.C., Renooij W., Murzilli S., Klomp L.W., Siersema P.D., Schipper M.E., Danese S. (2011). Farnesoid X receptor activation inhibits inflammation and preserves the intestinal barrier in inflammatory bowel disease. Gut.

[24] Degirolamo C., Modica S., Palasciano G., Moschetta A. (2011). Bile acids and colon cancer: Solving the puzzle with nuclear receptors. Trends Mol. Med..

[25] Gadaleta R.M., Garcia-Irigoyen O., Moschetta A. (2017). Bile acids and colon cancer: Is FXR the solution of the conundrum?. Mol. Asp. Med..

[26] Kim T.Y., Kim S., Kim Y., Lee Y.S., Lee S., Lee S.H., Kweon M.N. (2022). A High-Fat Diet Activates the BAs-FXR Axis and Triggers Cancer-Associated Fibroblast Properties in the Colon. Cell Mol. Gastroenterol. Hepatol..

[27] O’Keefe S.J., Li J.V., Lahti L., Ou J., Carbonero F., Mohammed K., Posma J.M., Kinross J., Wahl E., Ruder E. (2015). Fat, fibre and cancer risk in African Americans and rural Africans. Nat. Commun..

[28] Yokota A., Fukiya S., Islam K.B., Ooka T., Ogura Y., Hayashi T., Hagio M., Ishizuka S. (2012). Is bile acid a determinant of the gut microbiota on a high-fat diet?. Gut Microbes.

[29] Bajor A., Gillberg P.G., Abrahamsson H. (2010). Bile acids: Short and long term effects in the intestine. Scand. J. Gastroenterol..

[30] McGarr S.E., Ridlon J.M., Hylemon P.B. (2005). Diet, anaerobic bacterial metabolism, and colon cancer: A review of the literature. J. Clin. Gastroenterol..

[31] Vavassori P., Mencarelli A., Renga B., Distrutti E., Fiorucci S. (2009). The bile acid receptor FXR is a modulator of intestinal innate immunity. J. Immunol..

[32] Inagaki T., Moschetta A., Lee Y.K., Peng L., Zhao G., Downes M., Yu R.T., Shelton J.M., Richardson J.A., Repa J.J. (2006). Regulation of antibacterial defense in the small intestine by the nuclear bile acid receptor. Proc. Natl. Acad. Sci. USA.

[33] Miyazaki T., Shirakami Y., Mizutani T., Maruta A., Ideta T., Kubota M., Sakai H., Ibuka T., Genovese S., Fiorito S. (2021). Novel FXR agonist nelumal A suppresses colitis and inflammation-related colorectal carcinogenesis. Sci. Rep..

[34] De Gottardi A., Touri F., Maurer C.A., Perez A., Maurhofer O., Ventre G., Bentzen C.L., Niesor E.J., Dufour J.F. (2004). The bile acid nuclear receptor FXR and the bile acid binding protein IBABP are differently expressed in colon cancer. Dig. Dis. Sci..

[35] Maran R.R., Thomas A., Roth M., Sheng Z., Esterly N., Pinson D., Gao X., Zhang Y., Ganapathy V., Gonzalez F.J. (2009). Farnesoid X receptor deficiency in mice leads to increased intestinal epithelial cell proliferation and tumor development. J. Pharm. Exp..

[36] Modica S., Murzilli S., Salvatore L., Schmidt D.R., Moschetta A. (2008). Nuclear bile acid receptor FXR protects against intestinal tumorigenesis. Cancer Res..

[37] Selmin O.I., Fang C., Lyon A.M., Doetschman T.C., Thompson P.A., Martinez J.D., Smith J.W., Lance P.M., Romagnolo D.F. (2016). Inactivation of Adenomatous Polyposis Coli Reduces Bile Acid/Farnesoid X Receptor Expression through Fxr gene CpG Methylation in Mouse Colon Tumors and Human Colon Cancer Cells. J. Nutr..

[38] Leclerc D., Deng L., Trasler J., Rozen R. (2004). ApcMin/+ mouse model of colon cancer: Gene expression profiling in tumors. J. Cell Biochem..

[39] Su L.K., Kinzler K.W., Vogelstein B., Preisinger A.C., Moser A.R., Luongo C., Gould K.A., Dove W.F. (1992). Multiple intestinal neoplasia caused by a mutation in the murine homolog of the APC gene. Science.

[40] Fu T., Coulter S., Yoshihara E., Oh T.G., Fang S., Cayabyab F., Zhu Q., Zhang T., Leblanc M., Liu S. (2019). FXR Regulates Intestinal Cancer Stem Cell Proliferation. Cell.

[41] Cariello M., Peres C., Zerlotin R., Porru E., Sabba C., Roda A., Moschetta A. (2017). Long-term Administration of Nuclear Bile Acid Receptor FXR Agonist Prevents Spontaneous Hepatocarcinogenesis in Abcb4(-/-) Mice. Sci. Rep..

[42] Modica S., Petruzzelli M., Bellafante E., Murzilli S., Salvatore L., Celli N., Di Tullio G., Palasciano G., Moustafa T., Halilbasic E. (2012). Selective activation of nuclear bile acid receptor FXR in the intestine protects mice against cholestasis. Gastroenterology.

[43] Okayasu I., Ohkusa T., Kajiura K., Kanno J., Sakamoto S. (1996). Promotion of colorectal neoplasia in experimental murine ulcerative colitis. Gut.

[44] Melgar S., Karlsson L., Rehnstrom E., Karlsson A., Utkovic H., Jansson L., Michaelsson E. (2008). Validation of murine dextran sulfate sodium-induced colitis using four therapeutic agents for human inflammatory bowel disease. Int. Immunopharmacol..

[45] Kuipers E.J., Grady W.M., Lieberman D., Seufferlein T., Sung J.J., Boelens P.G., van de Velde C.J., Watanabe T. (2015). Colorectal cancer. Nat. Rev. Dis. Primers.

[46] Font-Burgada J., Sun B., Karin M. (2016). Obesity and Cancer: The Oil that Feeds the Flame. Cell Metab..

[47] Gadaleta R.M., Scialpi N., Peres C., Cariello M., Ko B., Luo J., Porru E., Roda A., Sabba C., Moschetta A. (2018). Suppression of Hepatic Bile Acid Synthesis by a non-tumorigenic FGF19 analogue Protects Mice from Fibrosis and Hepatocarcinogenesis. Sci. Rep..

[48] Kim I., Morimura K., Shah Y., Yang Q., Ward J.M., Gonzalez F.J. (2007). Spontaneous hepatocarcinogenesis in farnesoid X receptor-null mice. Carcinogenesis.

[49] Yang F., Huang X., Yi T., Yen Y., Moore D.D., Huang W. (2007). Spontaneous development of liver tumors in the absence of the bile acid receptor farnesoid X receptor. Cancer Res..

[50] Li S., Xu Z., Guo J., Zheng J., Sun X., Yu J. (2020). Farnesoid X receptor activation induces antitumour activity in colorectal cancer by suppressing JAK2/STAT3 signalling via transactivation of SOCS3 gene. J. Cell Mol. Med..

[51] Guo J., Zheng J., Mu M., Chen Z., Xu Z., Zhao C., Yang K., Qin X., Sun X., Yu J. (2021). GW4064 enhances the chemosensitivity of colorectal cancer to oxaliplatin by inducing pyroptosis. Biochem. Biophys. Res. Commun..

[52] Mauad T.H., van Nieuwkerk C.M., Dingemans K.P., Smit J.J., Schinkel A.H., Notenboom R.G., van den Bergh Weerman M.A., Verkruisen R.P., Groen A.K., Oude Elferink R.P. (1994). Mice with homozygous disruption of the mdr2 P-glycoprotein gene. A novel animal model for studies of nonsuppurative inflammatory cholangitis and hepatocarcinogenesis. Am. J. Pathol..

[53] Popivanova B.K., Kitamura K., Wu Y., Kondo T., Kagaya T., Kaneko S., Oshima M., Fujii C., Mukaida N. (2008). Blocking TNF-alpha in mice reduces colorectal carcinogenesis associated with chronic colitis. J. Clin. Investig..

